# Pseudohypoparathyroidism: phenotypic spectrum in kindred

**DOI:** 10.1186/1687-9856-2015-S1-P84

**Published:** 2015-04-28

**Authors:** Antonette A A Natino, Rashida Vasanwala

**Affiliations:** 1 Endocrinology & Diabetes Service, Department of Paediatrics, KK Women’s & Children’s Hospital Singapore

## 

Pseudohypoparathyroidism (PHP) encompasses a heterogeneous group of disorders due to an inactivating mutation in the GNAS gene which encodes the α subunit of G_s_ proteins (Gsα). Gsα plays a crucial role in intracellular signal transduction of peptides, hormones and neurotransmitter receptors in multiple tissues. Key features of PHP include Albright Hereditary Osteodystrophy (AHO) and biochemical evidence of multiple hormone resistances. There are several conflicting mechanisms for its heterogeneity; a possible explanation is a tissue-specific differential imprinting of Gsα protein. PHP type1a has AHO with multiple hormone resistance and is inherited as maternal imprinting defect. PHP type 1b presents with hormone resistance but no AHO features and is probably due to epigenetic methylation defects. Peudopseudohypoparathyroidism is another subtype with AHO but absence of hormone resistance and is inherited as paternal inactivating mutation. We describe a family with female members having PHP with variable clinical and biochemical features.

Two female siblings from non-consanguineous parents, patient A (15 year) & patient B (13 year) presented at birth with raised TSH levels and were diagnosed to have congenital hypothyroidism and treated with thyroxine replacement. Patient A was noted to have infantile obesity with short stature and evolving phenotypic features consistent with AHO. Further history revealed that her sister patient B has similar phenotypic appearance and was confirmed on clinical exam. Biochemical evaluation for Patient A showed a borderline low calcium and normal phosphate level with elevated PTH while Patient B had normal calcium and phosphate levels with high PTH. Both patients entered puberty at 11 years and patient A progressed to breast tanner 4 but has no menarche at 15 years despite pubertal gonadotropin levels (Table 1), advanced bone age (18 years) & mature uterus. Patient B is currently 12.5 years old and has progressed to tanner 3 breasts with no menarche. Mother exhibits features of AHO without hormone resistance. The father and younger brother do not have any features of AHO.

**Table 1:** 

	Patient A	Patient B	Mother
**AHO Features**			

Brachydactyly	+	+	+

Short stature	+ (136cm, - 4.05 SDS)	+ (134cm, -2.84 SDS)	+ (145cm, -2.79 SDS)

Obesity	+ (32.44 kg/m^2^,+2.04 SDS )	-	-

Round face	+	+	-

Ectopic ossification	-	-	-

Cognitive dysfunction with need for special school	+	+	+

**PTH** (0.9 -6.2pmol/L)	26.4	27.2	8.1

**Calcium** (2.3-2.63mmol/L)	2.19	2.35	2.31

**Phosphate** (1.0-1.8 mmol/L)	1.4	1.7	1.2

**FT4** (10.3-25.7pmol/L)	12.4	15.4	13.6

**TSH** (0.50-4.50 mIU/L)	8.05	8.36	3.42

**FSH** IU/L	7.7	9.5	

**LH** IU/L	8.92	5.01	

**Estradiol** pmol/L	87	169	

The two patients with PHP described above had an interesting presentation as congenital hypothyroidism with features of AHO evolving later in life. They exhibit clinical evidence of maternal transmission with end-organ resistance. This family illustrates heterogeneity of presentation of GNAS mutation.

Written informed consent was obtained from the patients for publication of this abstract. A copy of the written consent is available for review by the Editor of this journal.

